# Aging and selective sensorimotor strategies in the regulation of upright balance

**DOI:** 10.1186/1743-0003-4-19

**Published:** 2007-06-20

**Authors:** Nicoleta Bugnariu, Joyce Fung

**Affiliations:** 1 School of Rehabilitation Sciences, University of Ottawa, 451 Smyth Road, Room 3057, Ottawa, Ontario, K1H 8M5, Canada; 2 Jewish Rehabilitation Hospital CRIR Research Center, Laval, QC, H7V 1R2, Canada; 3 School of Physical and Occupational Therapy, McGill University, Montreal, QC, H3G 1Y5, Canada

## Abstract

**Background:**

The maintenance of upright equilibrium is essentially a sensorimotor integration task. The central nervous system (CNS) has to generate appropriate and complex motor responses based on the selective and rapid integration of sensory information from multiple sources. Since each sensory system has its own coordinate framework, specific time delay and reliability, sensory conflicts may arise and represent situations in which the CNS has to recalibrate the weight attributed to each particular sensory input. The resolution of sensory conflicts may represent a particular challenge for older adults given the age-related decline in the integrity of many postural regulating systems, including musculoskeletal and sensory systems, as well as neural processing and conduction of information. The effects of aging and adaptation (by repeated exposures) on the capability of the CNS to select pertinent sensory information and resolve sensory conflicts were thus investigated with virtual reality (VR) in the present study.

**Methods:**

Healthy young and older adults maintained quiet stance while immersed in a virtual environment (VE) for 1 hour during which transient visual and/or surface perturbations were randomly presented. Visual perturbations were induced by sudden pitch or roll plane tilts of the VE viewed through a helmet-mounted display, and combined with or without surface perturbations presented in a direction that was either identical or opposite to the visual perturbations.

**Results:**

Results showed a profound influence of aging on postural adjustments measured by electromyographic (EMG) responses and displacements of the center of pressure (COP) and body's center of mass (COM) in the recovery of upright stance, especially in the presence of sensory conflicts. Older adults relied more on vision as compared to young adults. Aging affects the interaction of the somatosensory and visual systems on the control of equilibrium during standing and the ability of CNS to resolve sensory conflicts. However, even with a one-hour immersion in VE and exposure to sensory conflicts, it is possible for the CNS to recalibrate and adapt to the changes, while improving balance capability in older adults.

**Conclusion:**

Preventive and rehabilitation programs targeting postural control in older adults should take into account the possible impairment of sensory organization or sensorimotor integration and include VE training under conditions of sensory conflicts.

## Background

The CNS processes information from multiple sensory channels, and adapts to the environment by generating task-specific and goal-directed movements. Unexpected movement of a support surface elicits rapid, automatic and coordinated postural responses that are triggered primarily by somatosensory afferents [1-4]. These responses are not merely segmental reflexes organized at the level of the spinal cord, but rather depend on the integration of proprioceptive, visual and vestibular information at many levels of the neuraxis [5-7]. Head-based sensors are mapped downwards from neck muscles to leg muscles, whereas somatosensory afferents from the feet and legs are mapped upwards to the trunk [8].

Visual inputs were once thought to be irrelevant to sudden stance perturbations, since the sensation of motion induced by moving visual fields has a relatively long latency [9]. Subsequent experiments have shown that visual information that conflicts with those arising from other sensory channels can have a rapid and profound effect on postural responses [10,11]. The influence of moving visual fields on postural stability depends on the characteristics of the visual environment, and of the support surface, including the size of the base of support, its rigidity or compliance [12,13]. Within physiological limits, a central recalibration process exists to produce appropriate responses even in the presence of sensory conflicts, i.e. when visual perception of the environment is discordant with proprioceptive information gathered from the support surface. Aging is associated with the decline in the integrity of many postural regulating systems [14-16], but its effect on the sensory inputs recalibration process remains to be determined. One question of interest is whether selective sensorimotor strategies necessary to maintain balance in the presence of sensory conflicts can be entrained.

There is a need for intense task-related practice to promote the re-acquisition of motor skills in rehabilitation. Virtual reality (VR) is a valuable tool for therapeutic interventions that require adaptation to complex, multimodal environments [13]. VR systems have been applied to the training of upper and lower extremities after stroke [17,18], to improve mobility in persons with impaired spatial abilities or to train balance control [19,20] and in vestibular rehabilitation [21]. Thus, the aim of the present study was to determine the effects of aging and repeated exposures on the capability of the CNS to select pertinent sensory information and resolve sensory conflicts created by VR.

## Methods

### Subjects

Ten young adults (5 males and 5 females of mean age 26 ± 5.1 y.o.) and 10 older adults (5 males and 5 females of mean age 72 ± 3.3 y.o.) participated in this study. Subjects were healthy with no neurological problems, musculoskeletal conditions or motion sickness. Informed consent to participate in the experiment previously approved by the institutional ethics committee was obtained from all subjects.

### Procedure

During quiet stance, subjects were exposed to random visual and/or surface perturbations consisting of ramp-and-hold tilts of 8° (peak velocity of 36°/s) in each direction of the pitch and roll planes. Visual perturbations were induced by sudden movements of a virtual environment (VE) viewed through a helmet-mounted display (HMD, Kaiser Optics ProView™ XL50, with a field of view of 50° diagonal, 30° vertical × 40° horizontal, weight 36 ounces). The VE consisted of a 3D rendered computer-simulated room generated by SoftImage XSI on a CAREN 2.3.0 Workstation (Motek Inc) and complete with windows, columns, flooring and ceiling textures. The perturbations consisted of: 1) **visual-only**, the VE was tilted but the surface was stationary; 2) **surface-only**, the surface was tilted but the VE was fixed; 3) **discordant**, visual perturbation was combined with synchronized surface perturbation in the same direction, and 4) **concordant**, visual perturbation was combined with synchronized surface perturbation in the opposite direction resembling the real life perception.

The support surface was mounted over a six-degree-of-freedom motion base servo-controlled by six electrohydraulic actuators [22]. The initial stance posture consisted of weight evenly distributed between feet placed on 2 force-plates (AMTI OR6-7), heels 15 cm apart and feet oriented in a 15° toe-out position. Subjects were instructed to maintain balance to the best of their abilities without taking steps where possible. If a step was taken, the subjects were instructed to resume the initial stance posture. A total of 72 perturbation trials were completed for a minimum of 1 hour VE immersion.

### Data recording

A six-camera VICON 512 system (Oxford Metrics) was used to capture 3D position data at 120 Hz from 36 retroreflective markers placed over anatomical landmarks and 4 markers placed on the movable platform. Ground reaction forces and moments from the force-plates were acquired at 1,080 Hz.

Eight bilateral muscles were instrumented with bipolar Ag-AgCl surface electrodes to record electromyographic (EMG) signals using a Noraxon system: tibialis anterior (TA), gastrocnemius medialis (MG), vastus lateralis (VL), semitendinosus (ST), tensor fascia latae (TFL), erector spinae (ES) at the L3 level, neck extensor (NE) and neck flexor sternocleidomastoideus (SCM). EMG signals were amplified, digitized, band-pass filtered (10–400 Hz low-pass) and sampled at 1,080 Hz. EMG signals were further full-wave rectified and lowpass filtered at 100 Hz during offline analysis. Functional balance and mobility in terms of gait velocity, ability to maintain tandem stance, timed repeated sit-to-stand, as described in a physical performance battery [23] were assessed before and after VE exposure and perturbation trials.

### Data analysis

A biomechanical model (Plug-In Gait) was used in conjunction with kinematic data and anthropometric measures to calculate the displacement of the body's center of mass (COM). Resultant center of pressure (COP) in the horizontal plane was calculated as the weighted sums from the vertical force and anteroposterior (A/P) and mediolateral (M/L) moments from the individual force plate as described previously [24]. Inertial components in forces and COP data due to movement of the support surface were corrected [25]. Muscle latencies were determined as the first burst that exceeded a threshold of two standard deviations above the background mean level and lasting at least 50 ms, with an activation probability of at least 50%. Data from different trials of each individual were ensemble-averaged across each one of the four testing conditions (vision only, surface only, discordant and concordant) and each one of the directions of pitch (toes-up/down) and tilt (left/right-down). These averages were pooled to produce a population average (young and old) for each direction of perturbations and condition of testing. To estimate the ability to adapt, average responses from the first 10 and last 10 trials were also calculated and compared.

## Results

### Kinematics

**Figure 1:**
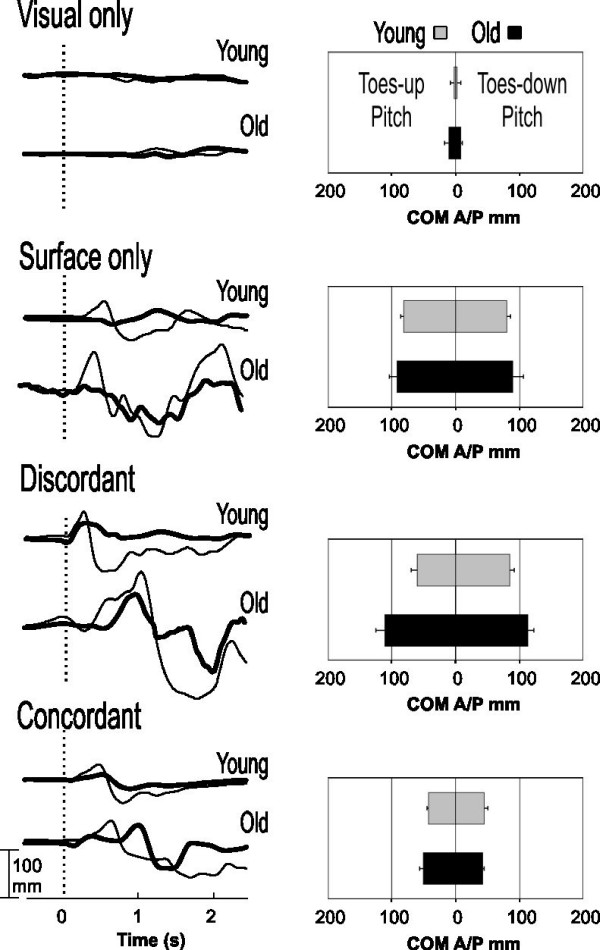
**Pitch plane COP and COM responses in different sensory conditions**. Representative example of individual traces of COP (thin lines) and COM (thick lines) from one young and one old subject (left panel) exposed to toes-up tilt of the surface. Bar graphs on the right panel show COM peak-to-peak excursions (mean ± S.D.) averaged across 10 young subjects (gray bars) and 10 older subjects (black bars) in both toes-up (left column) and toes-down (right column) directions.

**Figure 2:**
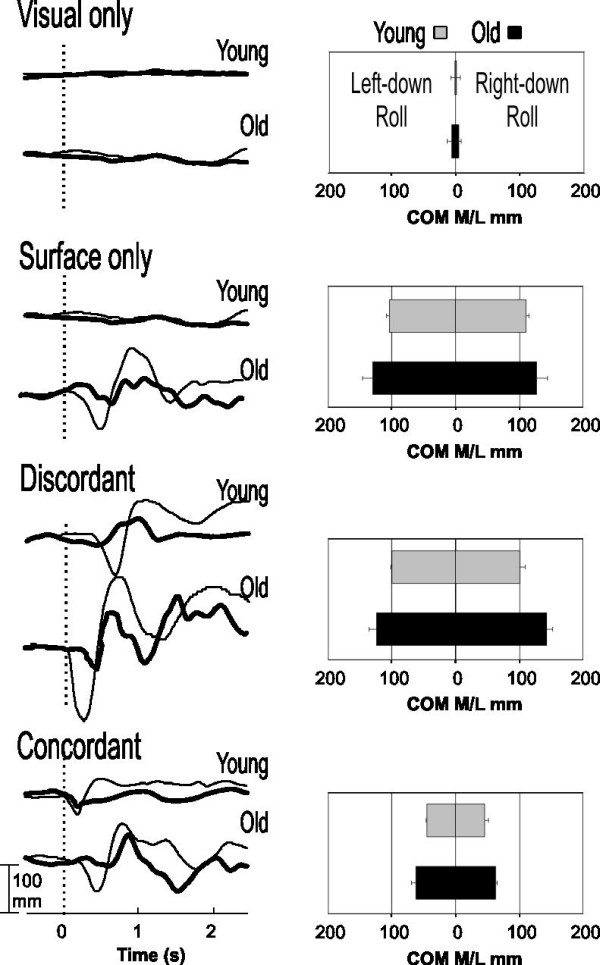
**Roll plane COP and COM responses in different sensory conditions**. Representative example of individual traces of COP (thin lines) and COM (thick lines) from one young and one old subject (left panel) exposed to right-down roll of the surface. Bar graphs on the right panel show COM peak-to-peak excursions (mean ± S.D.) averaged across 10 young subjects (gray bars) and 10 older subjects (black bars) in both left-down (left column) and right-down roll (right column) directions.

Representative examples of COP and COM traces (thin and thick lines, respectively, left-sided graphs), as well as group averages of COM (right-sided bar graphs) during pitch and roll perturbations are shown in Figures 1 and 2, respectively. During **visual-only **perturbations, minimal displacements of COP and COM were observed in both subject groups. Surface perturbations, with or without visual perturbations, provoked displacements of COP and COM first in the direction of the perturbation, and then reversed to oppose the perturbation for balance adjustment. Changes in COP always preceded and encompassed those of COM. In young adults, COP and COM displacements were smallest during perturbation where the visual and somatosensory stimuli were **concordant**. The displacements were markedly larger in older adults and took longer duration to return to the neutral positions. In older adults, during **surface-only **and **discordant **perturbations, COM and COP excursions increased substantially and took longer or never return to a neutral or stable position.

During **visual-only **perturbations, similar and minimal (2–10 mm) postural sway was observed in both the young (gray) and old (black) groups of subjects (bar graphs, Figures 1 and 2). In general, across all other conditions of testing, older adults displayed significantly larger COM peak-to-peak excursions (20 to 40 mm more than young adults). The presence of sensory conflicts in **discordant **perturbations, and to a certain extent, **surface-only **perturbations, induced significantly larger COM excursions than **concordant **perturbations in both young and old adults. However, the presence of sensory conflicts required a larger correction in older adults. During **surface-only **perturbations, mean COM excursions for older subjects compared to young were 10–15 mm larger in pitch (Figure 1) and 15–25 mm in roll (Figure 2) perturbations. During conditions of **discordant **perturbations, COM excursions were 30–50 mm and 20–40 mm larger in old subjects compared to young during pitch (Figure 1) and roll (Figure 2) perturbations. During conditions of **concordant **perturbations, COM excursions were not significantly different between old and young subjects. The average COP values (not shown) displayed similar trends, although always larger in range as compared to the COM.

### EMG activity

**Figure 3:**
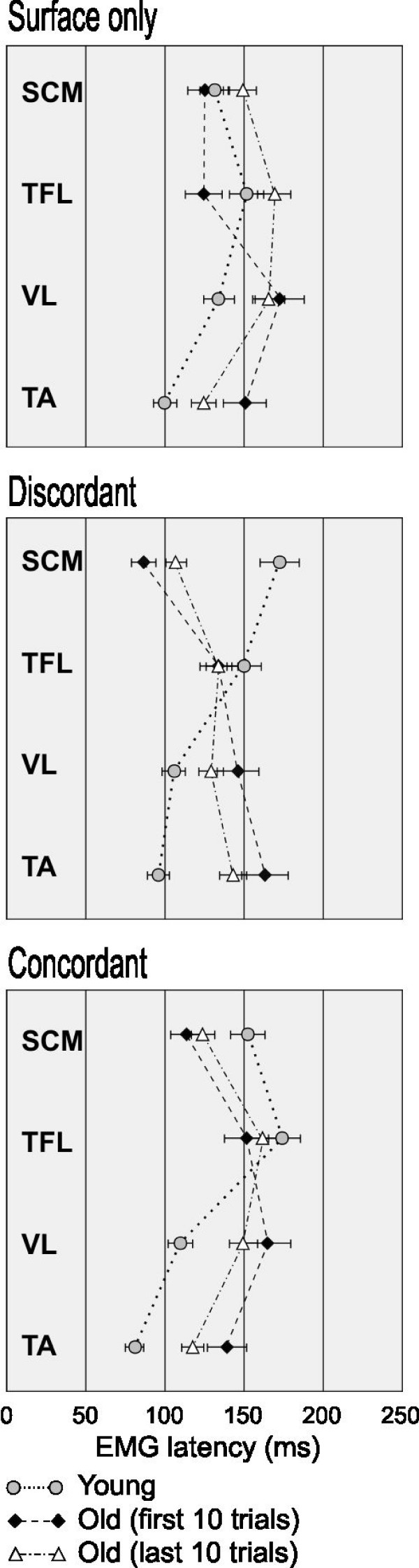
**Single-session adaptation of muscle responses**. EMG latencies (mean ± S.D.) of ventral muscles responding to toes-up pitch surface perturbations across young (gray circles) and old subjects. Note the decrease in the latencies in old subjects from the first 10 (black diamonds) to the last 10 (open triangles) trials.

The average EMG latencies of ventral muscles responding in the toes-up pitch direction during surface-only, discordant and concordant perturbations in young and old adults are shown in Figure 3. Young subjects showed similar latencies from the first 10 to the last 10 trials, and thus are averaged across all trials of similar conditions (Figure 3, gray circles). Old subjects showed increasingly earlier activations from the first 10 (Figure 3, black diamonds) to the last 10 trials (Figure 3, open triangles). No muscle activation was observed in young adults during visual-only perturbations. In old adults, sporadic muscles activations were present during visual-only perturbation but the activation probability of 50% was not reached, and thus were not included further in the analysis. In young adults, muscle recruitment generally followed a distal-to-proximal sequence, regardless of perturbation direction or sensory conflicts. The ankle muscles, TA and MG, were first to be activated during toes-up and toes-down tilt, respectively, at a latency of 80–100 ms. VL and ST were activated 110–130 ms, followed by TFL and ES approximately 30–50 ms later, while the neck muscles SCM and NE were activated at 150–170 ms.

In older adults, the distal-to-proximal sequence of EMG activation was less consistent, especially under sensory conflicts, during **surface-only **and **discordant **perturbations (Figure 3, black diamonds), where a reverse sequence was observed. In some older subjects, following discordant visual and somatosensory stimuli, activation of neck muscles preceded distal leg muscles by 25–70 ms. Generally, the EMG onset latencies of older adults, which were already delayed as compared to young adults, were further prolonged by 40–60 ms in conditions of sensory conflicts. However, adaptation occurred during the one-hour session such that ankle EMG latencies (Figure 3, open triangles) were 20 ms shorter in the last 10/72 perturbations as compared to the first 10 trials.

### Clinical measures

The average number of steps taken by older subjects also decreased from 3 during the first 10 trials to one in the last 10 trials. At the end of the 1 h immersion in the VE and repeated exposures to sensory conflicts perturbations, four old adults scored 1–2 points higher on their ability to maintain tandem stance. No change was observed in gait speed and timed repeated sit-to-stand. All subjects tolerated well the use of the HMD with the exception of one older subject who reported mild discomfort due to the tight fitting. All subjects were able to complete the 1 h immersion in the VE with no reports of nausea.

## Discussion

Conflicting visual and somatosensory stimuli modulate automatic postural responses in both healthy young and old adults but the presence of sensory conflicts had a larger impact on the selection of appropriate strategies for balance control in older adults. When the VE was manipulated to provide distorted visual perception, i.e. during surface-only or discordant perturbations, older adults took more steps, had longer EMG onset latencies as well as larger COP and COM excursions. Postural instability as measured by COM excursions increase markedly especially during discordant perturbations. Similar age-related postural instability was also reported by Mahboobin et al. [26] who showed that optic flow induced larger postural responses in old subjects than in subjects who had adapted from unilateral loss of vestibular function. It is plausible that delayed or diminished vestibular and somatosensory inputs in older adults increases their sensory thresholds to complex multimodal stimuli, thereby inducing a greater reliance on visual inputs and making it more difficult for them to respond selectively to visual and physical destabilization. Excessive reliance on visual input may be a natural compensatory strategy to cope with poor balance in seniors, but it can be problematic when the visual information is not reliable.

Visual-only perturbations elicit minimal postural responses in both young and old subjects with only sporadic activations of muscles in older adults, suggesting that under normal circumstances when there is a stable support surface, the somatosensory information is weighted more in regulating upright posture. The use of HMD to deliver the visual perturbation might have also limited the influence of visual inputs. Postural responses coupled with optic flow are less frequent when the optic flow is delivered in a central field of view, like the HMD, as compared to BNAVE display with a full field of view [21,27]. The influence of moving visual fields on postural stability depends on the characteristics not only of the visual environment, static vs. dynamic [28], but also of the support surface. Somatosensory information from the lower extremities and trunk is particularly important for maintaining balance when the subject maintains contact with a large, rigid, and stable support surface [7]. In such conditions, quiet stance is usually not destabilized by moving the visual field, except for children who are more visually dependent [29]. In contrast, surface-only perturbations present a postural challenge for both young and old. EMG onset latencies can be delayed and prolonged, while postural sway increases, as compared to concordant visual and somatosensory perturbations. Similar effects of visual stabilization were observed on initial bursts of ankle muscles [11].

Discordant perturbations are most challenging with longest onset latencies of neck muscles observed in young adults, suggesting that they deal with the mismatch in visual and somatosensory information by either attempting to suppress visual information altogether, or re-weight proprioceptive feedback with increasing reliance. Older adults adopted a completely opposite strategy by activating the neck muscles first, suggesting an excessive reliance on visual inputs or the need to for head stabilization.

With repeated exposures to VR-induced sensory conflicts, a general training effect associated with less stepping responses and improved ability to maintain balance was observed in older adults. The ability to anticipate the physical constraints of the environment and adapt the balance behaviour accordingly is the result of intricate sensorimotor integration. The cognitive processes range from correctly perceiving and interpreting information from different body sensors (somatosensory, vestibular and visual) to planning and coordinating the effectors appropriately to produce the desired movement. Motor learning is promoted by factors such as changing environmental contexts, alterations in the physical demands, problem solving, and random presentation of practice tasks, sufficient practice and patient empowerment [30]. Even with a one-hour immersion in VE and exposure to sensory conflicts, it seems possible for the CNS to recalibrate and adapt to the changes and improve balance capability in older adults. A training program of longer durations is needed to confirm sustainable long-term effects. Preventive and rehabilitation programs targeting postural control in older adults should take into account the possible impairment of sensory organization or sensorimotor integration, and consider VE training under conditions of sensory conflicts as a potential rehabilitation strategy.

## Conclusion

Conflicting visual and somatosensory stimuli can modulate automatic postural responses in both healthy young and old adults. Aging affects the interaction of the somatosensory and visual systems on the ability of the CNS to resolve sensory conflicts and to maintain upright stance equilibrium. Therefore, rehabilitation programs targeting postural control in seniors should take into account the possible impairment of sensory organization and consider the inclusion of exercises performed under conditions of sensory conflicts.

## Competing interests

The author(s) declare that they have no competing interests.

## Authors' contributions

NB has been involved in the conception and design, data acquisition, analysis and interpretation, as well as drafting the manuscript. JF participated in the design of the study and data interpretation, and critically revised the manuscript for its intellectual content. Both authors have read and approved the final manuscript.

## References

[B1] Diener HC, Horak FB, Nashner LM (1988). Influence of stimulus parameters on human postural responses. J Neurophysiol.

[B2] Inglis T, Macpherson JM (1995). Bilateral labrynthectomy in the cat: effects on postural response to translation. J Neurophysiol.

[B3] Runge CF, Shupert CL, Horak FB, Zajac FE (1998). Role of vestibular information in initiation of rapid postural responses. Exp Brain Res.

[B4] Fung J, Hughey L (2005). Postural responses triggered by multidirectional leg litfs and surface tilts. Exp Brain Res.

[B5] Fung J, Macpherson JM (1999). Attributes of quiet stance in the chronic spinal cat. J Neurphysiol.

[B6] Macpherson JM, Fung J (1999). Weight support and balance during stance in the chronic spinal cat. J Neurophysiol.

[B7] Horak FB, Macpherson JM, Davis FA (1996). Postural orientation and equilibrium. Handbook of Physiology: Section 12: Integration of Motor, Circulatory, Respiratory and Metabolic control during Exercises New York.

[B8] Mergner T, Huber W, Becker W (1997). Vestibular-neck interaction and transformation of sensory coordinates. J Vestib Res.

[B9] Nashner L, Berthoz A (1978). Visual contribution to rapid motor responses during postural control. Brain Res.

[B10] Vidal PP, Berthoz A, Millanvoye M (1982). Difference between eye closure and visual stabilization in the control of posture in man. Aviat Space Environ Med.

[B11] Keshner EA, Kenyon RV, Langston J (2004). Postural responses exhibit multisensory dependencies with discordant visual and support surface motion. J Vestib Res.

[B12] Streepey JW, Kenyon RV, Keshner EA (2006). Field of view and base of support width influence postural responses to visual stimuli during quiet stance. Gait Posture.

[B13] Keshner EA, Kenyon RV (2004). Using immersive technology for postural research and rehabilitation. Assistive Technology.

[B14] Horak F, Shupert C, Mirka A (1989). Components of postural dyscontrol in the elderly: a review. Neurobiol Aging.

[B15] Manchester D, Woollacott M, Zederbauer-Hylton N, Marin O (1989). Visual, vestibular and somatosensory contributions to balance control in the older adult. J Gerontol.

[B16] Maki BE, McIlroy WE (1996). Postural control in the older adult. Clin Geriatr Med.

[B17] Holden M, Todorov E, Callaban J, Bizzi E (1999). Virtual environment training improves motor performance in two patients with stroke. Neurology Report.

[B18] Deutsch JE, Latonio J, Burdca GC, Boian R (2001). Post-stroke rehabilitation with Rutgers ankle system A case study. Presence.

[B19] McComas J, Sveistrup H (2002). Virtual reality application for prevention, disability awareness and physical therapy rehabilitation in neurology: our recent work. Neurol Report.

[B20] Sveistrup H (2004). Motor rehabilitation using virtual reality. J Neuroengineering Rehabil.

[B21] Whitney SL, Sparto PJ, Brown K, Furman JM, Jacobson JL, Redfern MS (2002). The potential use of virtual reality in vestibular rehabilitation: Preliminary findings with the BNAVE. Neurology Report.

[B22] Henry S, Fung J, Horak FB (1998). Control of stance during lateral and anterior/posterior surface translations. IEEE Trans Rehab Eng.

[B23] Fung J, Johnstone E (1998). Lost in space. Multi-axial and multi-dimensional surface perturbations delivered by a novel motion base device [abstract]. Soc Neurosci.

[B24] Guralnik JM, Ferrucci L, Pieper CF (2000). Lower extremity function and subsequent disability: consistency across studies, predictive models, and value of gait speed alone compared with the short physical performance battery. J Gerontol A Biol Sci Med Sci.

[B25] Preuss R, Fung J (2004). A simple model to estimate force plate inertial components in a moving surface. J Biomechanics.

[B26] Mahboobin A, Loughlin PJ, Redfern MS, Sparto PJ (2005). Sensory re-weighting in human postural control during moving-scene perturbations. Exp Brain Res.

[B27] Sparto PJ, Redfern MS, Jasko JG, Casselbrant ML, Mandel EM, Furman JM (2006). The influence of dynamic visual cues for postural control in children aged 7–12 years. Exp Brain Res.

[B28] Amblard B, Cremieux J, Marchand AR, Carblanc A (1985). Lateral orientation and stabilization of human stance: static versus dynamic visual cues. Exp Brain Res.

[B29] Woollacott M, Sveistrup H (1992). Changes in the sequencing and timing of muscle response coordination associated with developmental transitions in balance abilities. Human Movement Sci.

[B30] Winstein CJ (1991). Knowledge of the results and motor learning: implications for physical therapy. Physical Therapy.

